# Community Mobilization and Community Incentivization (CoMIC) Strategy for Child Health in a Rural Setting of Pakistan: Study Protocol for a Randomized Controlled Trial

**DOI:** 10.3390/mps6020030

**Published:** 2023-03-13

**Authors:** Jai K. Das, Rehana A. Salam, Arjumand Rizvi, Sajid B. Soofi, Zulfiqar A. Bhutta

**Affiliations:** 1 Institute for Global Health and Development, Aga Khan University, Karachi 74800, Pakistan; 2 Melanoma Institute Australia, University of Sydney, Wollstonecraft, NSW 2065, Australia; 3 Centre of Excellence in Women and Child Health, Aga Khan University, Karachi 74800, Pakistan; 4 Centre for Global Child Health, The Hospital for Sick Children, 686 Bay Street, Toronto, ON M5G 0A4, Canada

**Keywords:** diarrhea, pneumonia, infection, community-based, incentive

## Abstract

Despite the decline in under-five mortality by over 60% in the last three decades, majority of child mortality is still attributable to communicable and infectious diseases that are not only preventable, but they are also treatable. We evaluated the potential impact of a participatory community engagement and innovative community incentivization (C3I) strategy for improving the coverage of child health interventions in a rural setting in Pakistan. We first undertook formative research to assess community knowledge and the likelihood of collective community strategy and conditional incentives for improving existing preventive and care-seeking practices for childhood diarrhea and pneumonia. We developed options for community incentivization and improving group practices, taking local norms and customs into account in the design of the community mobilization strategies and messages. These interventions were then formally evaluated prospectively in a three-arm cluster randomized controlled trial. Clusters were randomly assigned by a computer algorithm using restricted randomization by an external statistician (1:1:1) into three groups: community mobilization and incentivization (CMI); community mobilization only using an enhanced communication package (CM); and control group. The C3I was an innovative strategy as it involved serial incremental targets of collective improvement in community behavior related to improvement in the coverage of a composite indicator of fully immunized children (FIC), oral rehydration salt (ORS), and the sanitation index (SI). The evaluation was done by an independent data collection and analysis team at baseline and end line (after 24 months).

## 1. Background

In the year 2020, an estimated five million children under the age of five years died globally, without increased mortality attributable to COVID-19 [1]. Despite a drastic decline of 61% in under-five mortality in the last three decades, a majority is still attributable to communicable and infectious diseases, with pneumonia, diarrhea, and malaria continuing to remain the leading causes of death. This burden of under-five mortality is disproportionately concentrated in the poorest regions of the world, including South Asia and sub-Saharan Africa, with Nigeria, India, and Pakistan being the top three countries [2]. The major reasons include a lack of access to essential interventions and a lack of awareness. The COVID-19 pandemic with the ensuing lockdown strategies further restrained access and utilization of health services, including immunizations [3].

Diarrhea and pneumonia are regarded as diseases of poverty and are associated with undernutrition, poor hygienic conditions, and lack of awareness and access to healthcare [4]. These two diseases are considered to be forgotten epidemics, despite the existing enormous death toll, as in 2018, pneumonia was responsible for 800,000 and diarrhea for 437,000 under-five deaths globally [1]. Approximately 80% of these deaths occurred in low- and middle-income countries (LMICs), with 50% taking place in India, Nigeria, Pakistan, and Ethiopia alone [5].

In 2013, the Integrated Global Action Plan for Pneumonia and Diarrhea (GAPPD) launched by the World Health Organization (WHO) and UNICEF, called for an integrated approach to protect, prevent, and treat diarrhea and pneumonia. The GAPPD highlights the importance of protective and preventive interventions, such as exclusive breastfeeding (EBF) for six months, timely complementary feeding, vitamin A supplementations, immunization, care seeking, and improved water, hygiene, and sanitation conditions, in ending preventable child deaths caused by these diseases by 2025 [6]. In 2015, the United Nations adopted the Sustainable Development Goals (SDGs), which aimed to promote healthy lives and well-being for all children, with SDG-3 especially focusing on reducing under-5 mortality by 2030 [7].

Pakistan is amongst the top three countries for under-five deaths [8,9], with pneumonia and diarrhea being the leading cause of post-neonatal deaths, and this is despite existing interventions to prevent, manage and treat them, and an existing extensive primary and secondary health system with a well-developed community health workers program [10]. The coverage of essential child health interventions remains low; in 2017–2018, the Pakistan Demographic and Health Survey (PDHS) 2017–2018 [11] shows the prevalence of diarrhea among children under five as 19% and the prevalence of acute respiratory infection (ARI) as 14%. Notwithstanding the increase in care-seeking, as 71% seek care for diarrhea and 84% seek care for ARI, Pakistan has lower coverage for preventive and therapeutic interventions: the coverage of early breastfeeding initiation within one hour of birth is 20%, EBF for six months is 48%, timely complementary feeding for between six to eight months is 54% [11], vaccine coverage for children aged 12 to 23 months is 66%, 37% of children with diarrhea have been given ORS, only 8% have received zinc with ORS, and 46% have been given antibiotics for ARI. Hence, the coverage of all these interventions is well below the global targets [11,12].

The uptake of current interventions remains low, as they rely on health-related behaviors that are often resistant to change and are influenced by a variety of personal, cognitive, economic, social, cultural, and structural factors [13]. Over the past few decades, there has been a greater understanding of health-related behaviors, both at individual and community levels, and simultaneously, the use of formative research has also enhanced the implementation of culturally and geographically relevant programs [14]. Various forms of incentives (including user fees reduction, vouchers, and conditional and unconditional cash transfers) have been evaluated for their effectiveness on care seeking for child health, participation in health education, and health care visits, and have shown the potential to improve coverage of evidence-based interventions by targeting poverty alleviation and reducing barriers to accessing health care [15,16,17,18,19,20,21].

The Community Mobilization and Community Incentivization (CoMIC) trial was designed to implement and assess the effectiveness of a customized community mobilization and innovative incentivization strategy in improving adherence to evidence-based interventions for childhood diarrhea and pneumonia in a rural district of Pakistan. The CoMIC trial is a unique strategy since it did not involve conventional incentives like cash transfers, vouchers, or social insurance at an individual level; but proposed conditional incentives at a collective community level to improve health behaviors at a community level. This study first aimed to identify the baseline care-seeking practices, barriers, and facilitators through formative research, followed by the promotion of specific messages and strategies to improve the uptake of essential interventions for childhood diarrhea and pneumonia.

## 2. Conceptual Framework

Behavior change approaches aim to promote lifestyle changes; however, the evidence on the effectiveness of behavior change interventions/approaches is not ubiquitous [22]. Behavior change interventions can take various approaches, such as social change strategies, provision of facilities/supplies, or mass media/communication campaigns [23]. These approaches aim to provide education to the community to adopt a healthier lifestyle through innovative and interactive communication techniques, such as persuasion, counseling, motivation, etc. However, these techniques do not always guarantee a change within a community because the community or people do not only resist change, they resist the act of being changed.

**Figure 1:**
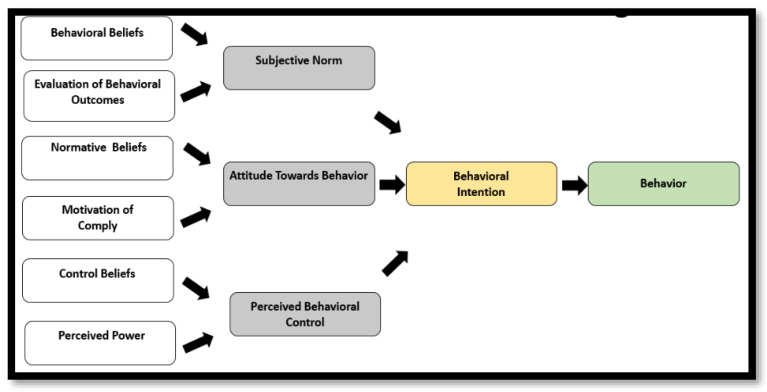
Theoretical Model of the CoMIC trial, based on the Theory of Reasoned Action (TRA) and the Theory of Planned Behavior (TPB).

We adopted an integrated behavioral model of the Theory of Reasoned Action (TRA) and the Theory of Planned Behavior (TPB), with slight modifications (Figure 1) [24,25,26]. This model assists in the understanding of beliefs that lead to behavioral intentions and consequent behaviors. The model helps us understand how subjective norms, attitudes towards behavior, and perceived behavioral control lead to a behavior intention [24,27,28,29]. TPB and TRA have been successful in predicting a wide range of health behaviors, such as drinking, smoking, exercise, health service utilization, breastfeeding, etc.

The framework first describes behavioral intentions as important determinants of behavior and then the constructs of the framework are:

- Theory of planned behavior—is useful in getting information on what details people need before attempting a behavior change.
- Subjective norms—these relate to a person’s belief about what others think that he or she should do.
- Attitudes towards behavior—these are determined by a belief that desired outcomes will occur if a particular behavior is followed.
- Perceived behavior control—recognizes that a behavior change is likely if a person has greater personal control over behavior (self-efficacy).

Using the health model theories, we aimed to influence the health behaviors of the communities living in the rural villages of the Tando Muhammad Khan (TMK) district in Sindh, Pakistan. This project was aimed at creating awareness, empowering the community to take their decisions, and also deciding on the short-term benefits that they can achieve through an innovative conditional collective community-based incentive (C3I) conditioned on serial incremental targets.

## 3. Methodology

### 3.1. Objective

The objective of the trial was to assess the impact of community engagement and demand creation strategy, involving a C3I, on uptake and adherence to recommended preventive and curative practices for diarrhea and pneumonia among children under the age of five years compared to standard care in a rural setting of Pakistan.

### 3.2. Study Setting

The study was conducted in the rural district of TMK in the province of Sindh, Pakistan. TMK has an area of 1814 km^2^ and a population of approximately 677,228 residents [11]. It comprises three administrative talukas and 17 Union Councils (UC), which is the lowest administrative unit. The district has a population density of approximately 75.8/km^2^ and an annual population increase of about 2.3%. TMK is the highest poverty level district (89.3%) in Sindh [30], as education and employment opportunities are limited and 62% of children are not enrolled in schools [30]. There are 15 basic health units (BHU), 20 government dispensaries, three rural health centers (RHC), and a district hospital in TMK. Over 43% of the population comprises children aged 0–14 years. The community residing in rural TMK belongs to a low socio-economic background with limited access to basic amenities.

### 3.3. Study Design

**Figure 2:**
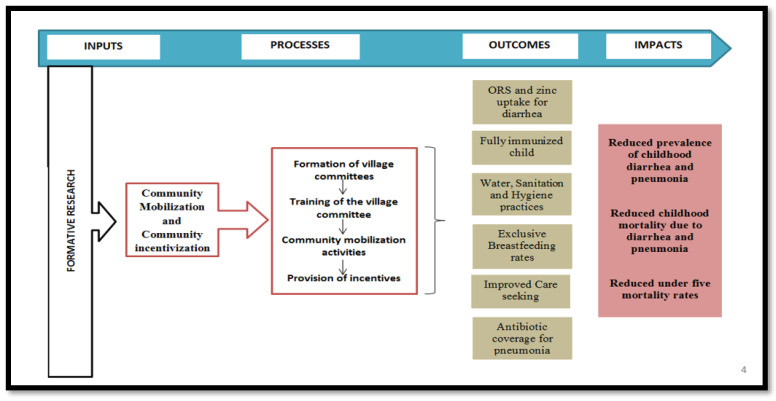
Design of the CoMIC trial.

The CoMIC trial was a prospective cluster randomized controlled trial (cRCT) with two intervention arms and a control arm (1:1:1) (Figure 2), and each cluster was based on a group of villages that were selected based on their geographic proximity and coherent and consistent communities.

### 3.4. Study Population

The population of this study included all households with at least one child under the age of five years who were permanent residents of the selected clusters of TMK.

### 3.5. Study Duration and Registration

The intervention was delivered over a period of two years and the trial was registered on clinicaltrial.gov (accessed on 23 December 2022) with trial registration number NCT03594279 [31].

### 3.6. Outcomes

The primary outcomes included:

1. Fully immunized child (FIC)—defined as vaccination determined by the age of the child and the vaccines received up to 23 months of age.
2. ORS use—defined as children who used ORS for the last episode of diarrhea.
3. Sanitation index (SI)—The SI was adopted from Webb et al. [32] and comprised four indices with a total of 15 items. These four indicators included the drinking water index (DWI), food index (FI), personal hygiene index (PHI), and domestic household hygiene index (DHI) (Table 1). Each item was scored as 0 or 1, with 1 representing a positive behavior. The indices were calculated as the sum of the items and SI was calculated as the sum of the four individual indices. For our study, we excluded PHI, as it would be difficult to assess with intermittent surveys, and the potential absence of subjects at home at the time of the survey. In our study, the SI comprised three indices with 12 items, hence a total score of 12.

The secondary outcomes:

1. EBF: EBF was defined as no other food or drink except breast milk (including milk expressed or from a wet nurse) taken for 6 months of life, but allowing the infant to receive ORS, drops, and syrups (vitamins, minerals, and medicines) [33].
2. Prevalence of diarrhea: Diarrhea was defined as the passage of three or more loose or liquid stools per day (or more frequent passage than is normal for the individual) within two weeks of the day of survey [34].
3. Prevalence of ARI: ARI was defined as children under 5 years of age, who have cough and/or difficulty breathing, with or without fever within two weeks of the day of survey [35].
4. Care seeking for childhood diarrhea and ARI: Healthcare-seeking behavior was defined as professional help sought from health-care services, health-care providers and/or community health workers for childhood illnesses.
5. Open defecation rates.

### 3.7. Formative Phase

We conducted a formative phase to first identify the existing socio-demographic dynamics, practices, and behaviors, along with context-specific barriers and facilitators for behaviors (immunization, hygiene, nutrition, care-seeking) related to childhood diarrhea and pneumonia and to assess the feasibility of the proposed intervention. Findings from the formative phase were then utilized to design a geographically and culturally relevant community-based mobilization and incentivization intervention. The formative phase comprised the geographic information system (GIS) mapping of the study area; a baseline household survey; focus group discussions (FDGs), and in-depth interviews (IDIs) with key stakeholders. These activities were conducted to:

- Assess the geographic spread and density of the population and the location of key landmarks.
- Assess the socio-demographic status and the major tribes within each village.
- Assess the existing health and hygiene-related practices and behaviors.
- Identify the enablers and barriers associated with the existing care-seeking practices, and uptake of evidence-based interventions for childhood diarrhea and pneumonia.
- Identify community preferences pertaining to the community mobilization activities and the development of Information, Education and Communication (IEC) material.
- Design the potential conditional community-based non-cash incentives that could be beneficial for the community.

### 3.8. Sample Size Calculation

Clusters were based on villages to prevent contamination. Findings from the baseline survey data were used to calculate the sample size, which was calculated for each of the primary outcomes with a power of 80% and an assumed relative improvement of 50% from baseline. From the baseline survey, ORS use for diarrhea was 47.6%; FIC coverage was 20.4%; while the mean WASH score was 5.63 (±1.7). The minimum sample size calculated was 13 clusters in each arm, but we included a total of 48 clusters with 16 clusters in each arm to provide sufficient power and cater to any unforeseeable adversities during the duration of the trial.

For the baseline/endline survey, the sample size was calculated to assess the effectiveness of the intervention. This was to detect at least a 25% conservative improvement from the baseline indicators of the primary outcome, assuming a design effect of 1.5, and to provide 80% power at the 5% level of significance with 10% attrition. A sample size of 3828 households (1276 per group) was calculated for the endline survey with estimated 13.5% households with at least one child less than five years of age and 5.44% households with at least one children less than two years of age.

### 3.9. Randomization

**Figure 3:**
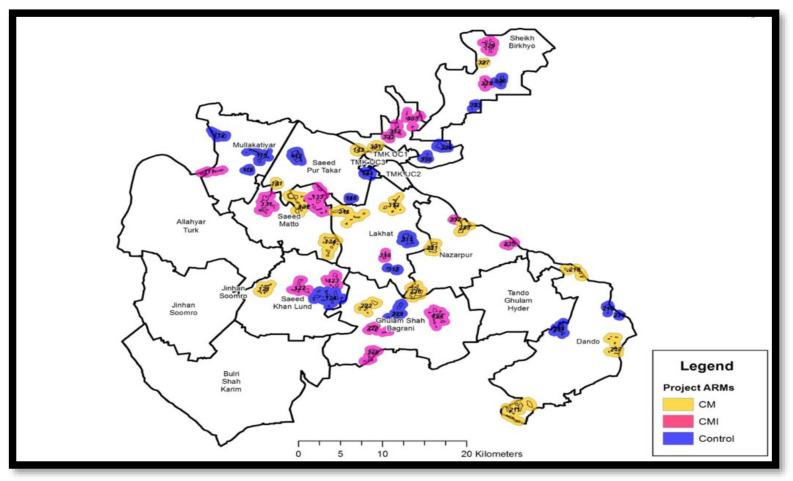
Geographic dispersion of clusters by the three study groups. CM: Community Mobilization; CMI: Community Mobilization and Incentivization.

The data from GIS in the formative phase was used to make these clusters by grouping villages in geographic proximity and with consistent and coherent communities, so that the population of each cluster was between 2000–3000. The unit of randomization was village/s (clusters) and this was performed through a computer-generated randomization sequence and allocation created by an independent statistician. The clusters were randomly assigned (1:1:1) to each of the two interventions and one control arm (Figure 3). A total of 16 clusters were randomly assigned through a computer-generated list to each group, adding up to a total of 48 clusters for the trial. Randomization was performed taking into consideration all the primary and secondary outcomes, socio-economic status, and education, and ensured at least a 10 km distance between individual clusters.

## 4. Intervention

### 4.1. Intervention Arm 1: Community Mobilization (CM)

The clusters in the community mobilization (CM) arm were involved in awareness and motivational activities throughout the intervention duration. Village committees (VC) were formed separately for male and female members in each of the 16 clusters and were responsible for carrying out CM activities. VCs comprised 6–8 members, including UC members, local elders/elites, religious leaders, and prominent male and female members of the community. The VC members were a diverse group of people with varying qualifications; and they also named their respective VCs to enhance association, identity, and affiliation.

#### 4.1.1. Training

All the VCs underwent a comprehensive six-day training on specific messages regarding the prevention and management of childhood diarrhea and pneumonia, to assist them in planning and carrying out awareness and motivational activities. The training was in simple language and avoided the use of any jargon. Following the training, the VCs facilitated one-hour group meetings in every village of their catchment area focusing on issues pertaining to childhood nutrition, WASH, vaccines, management of diarrhea and pneumonia, and encouraging community participation, and were supported by research staff.

#### 4.1.2. Information, Education and Communication (IEC) Materials

Findings from the formative phase guided the development of educational materials. Video messages were highlighted to be an effective means of communication in the formative phase. We developed the following materials.

- Posters.
- Pictorial brochures/flip charts.
- Short promotional videos in the local language (Sindhi) (58).

IEC materials were distributed, and promotional videos were played during the community sessions with an emphasis on the key messages.

#### 4.1.3. Activities

The formative phase suggested that the community was keen on mobilization and participatory learning approaches, with children being ‘agents of change’. The community awareness activities were held for males and females separately and for children:

- VC meetings (*Triggering sessions*).
- Meetings supervised by research staff (*Participatory Learning*).
- Sessions with children in schools/community (*Change Leaders*).

These activities were conducted to identify community-level health, nutrition, and sanitation problems, and to find locally feasible strategies to address them. The sessions focused on educating the community on the themes of WASH, breastfeeding, nutrition, immunization, childhood diarrhea, and ARI. They focused on encouraging community participation, identifying local challenges, and working together to achieve feasible solutions (Table 2). These group sessions were complemented by sessions from the research team where they discussed specific issues and solutions. The meeting schedule was as follows:

- Every month for the first six months.
- Every two months for the second six months.
- Quarterly in the second year of the study.

Separate regular sessions were also conducted in schools and the community for the children covering the following educational and physical activities:

- Educational sessions with key WASH and health-related massages.
- Display of IEC material inside school areas and classrooms.
- Involving schoolteachers to check personal hygiene—clothes, nails, shoes, and hair.
- School (playground, classroom, water containers) cleaning activities.
- Competitions and games related to hygiene and nutrition including posters competition, singing, etc.
- Gifts for the best student.

#### 4.1.4. Compliance

Compliance was checked by marking regular attendance and capturing geo-tagged pictures, which were routinely checked by staff. VCs maintained records, including meeting minutes, funds collection, and utilization.

### 4.2. Intervention Arm 2: Community Mobilization and Incentivization (CMI)

In addition to all the activities in Intervention Arm 1, clusters in the CMI Arm were provided with C3I. The C3I in the CoMIC trial was a novel incentive scheme that focused on the broader health and well-being of the community. It aimed to make the community work together and provided serial incremental targets to qualify for serial incentives, with the goal of improving behaviors aligned with the objective of this trial related to childhood diarrhea and pneumonia.

To establish eligibility for the C3I, a composite indicator was devised, comprising of the following three indicators (equal weightage), which were the primary outcomes of the trial:

- FIC (age-appropriate).
- ORS use for diarrhea.
- Sanitation index (which is a score based on DWI, FI, and DHI).

Each of the 16 clusters in the CMI group was asked to improve the above indicators over time (from baseline) to qualify for an incentive. Eligibility for the incentives was assessed at 6 months, 15 months, and 24 months, and incentives were given to each cluster that qualified at these three time points. Hence, each cluster could receive a maximum of three rounds of incentives throughout the duration of the intervention. Eligibility was assessed on improvements in the composite coverage as follows:

- At 6 months—10% improvement in the composite coverage from baseline (min. 5% each).
- At 15 months—25% improvement in the composite coverage from baseline (min. 15% each).
- At 24 months—50% improvement in the composite coverage from baseline (min. 30% each).

Independent intermediate surveys were conducted and the clusters in the CMI group, which showed improvement in the indicators according to the preset criteria, were eligible to receive incentives. The exact incentives were also decided by the individual VCs and based on need assessment and prioritization by the community themselves, and included structural benefits linked to health, including tube wells, water supply, toilets, water storage facility, or any other incentive as decided with the respective VCs. The total cost of these incentives was shared by the project (75%) and the community (25%) to improve ownership. The following incentives were identified as the top priorities in the formative phase.

1. Drinking water facilities (including simple handpumps, lead line handpumps, lead line water facilities with solar motor pumps).
2. Sanitation facilities–latrine/washing facility (complete structure), latrine/washing facility (only sub-structure), repair of the drainage system.

**Figure 4:**
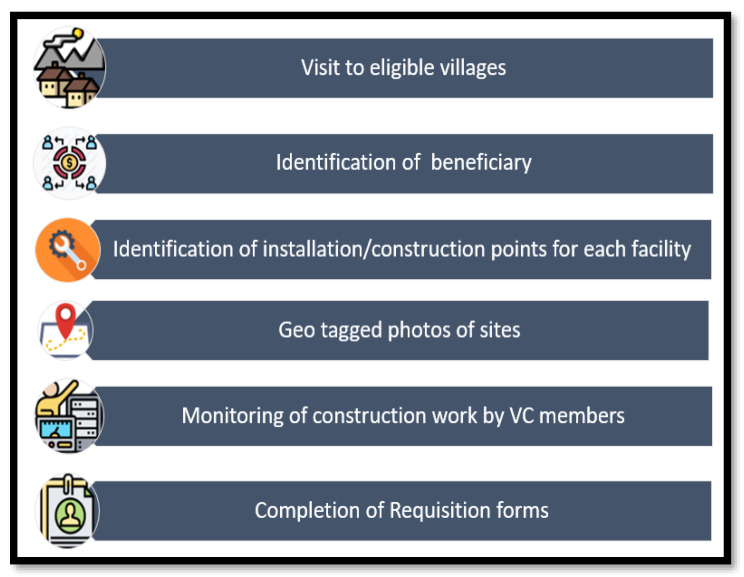
Summary of need assessment, prioritization, and incentive Delivery.

Once the incentives and beneficiaries were finalized, the research team and VCs visited the eligible villages and planned the location of the incentive facility (hand pump or toilet) on the premises. The VCs supervised all activities until the construction work was completed. The research team captured geo-tagged photographs of the sites on an ongoing basis. The process of need assessment, prioritization, and incentive distribution is summarized in Figure 4.

### 4.3. Control Arm: Standard Care

Control clusters continued to receive routine diarrhea and pneumonia management according to the existing standard of care.

## 5. Data Collection

All the data was collected by an independent team, which was not part of the core CoMIC team. These teams conducted the two interim surveys (at 6 and 15 months) to assess the eligibility for the incentives in the CMI group and were responsible for conducting the endline survey in all 48 clusters to assess the effectiveness of the intervention. The analysis team was blinded to the study groups.

### 5.1. Components of Data Collection

A line-listing was carried out in each cluster and a list of randomized households in each cluster was generated using an independent program, and 85 households were randomized in each cluster. Data were collected for the most recent diarrhea or/and ARI episode if there were multiple children who had diarrhea in the last 14 days in a single household. For IYCF indicators, data on the youngest child in the household (<2 years of age) were collected. For immunization, we collected data for all children less than two years of age. For SI, two spot checks were performed by different observers

The household survey data collection tool was developed using existing standardized tools for similar surveys, including the Demographic Health Survey (DHS) and the Multiple Indicator Cluster Survey (MICS). The questionnaire was modified where needed, translated into the local language (Sindhi), back-translated, and shared with managers for review and feedback. Face validity and construct validity for all the questionnaires were conducted with a small expert group and the refinement of questionnaires was completed based on expert feedback. The tool was pre-tested during staff training, modified, and shared for final approval. The data collection tool comprised various sections covering socio-demographic, health, and nutrition information (Table 3). Household information was obtained from the mother/caregiver of child under the age of five years.

Quantitative data was collected using handheld devices (Samsung tablets running Android 5.1). A customized application was developed using Java with MySQL & SQLite backend for data storage. Range and consistency check and skip patterns were built into the program to minimize the entry of erroneous data. If there were any inconsistencies identified, data collectors were contacted for rectification. For each child, a code was generated by the android application to maintain anonymity. All data were kept securely behind firewalls and fully anonymized prior to analysis.

### 5.2. Training

A total of four field teams were recruited, with each team comprising three female data collectors and one male team leader, considering the cultural sensitivities of the community. These data collectors were all graduates in various disciplines and had 14–16 years of education. Data were collected at baseline, six months (1st Interim), 15 months (2nd Interim), and 24 months (endline) post-intervention. A seven-day training workshop was organized by the core study team to train the field staff. The training focused on conceptual clarity of the data collection tools, operational procedures, data collection methods, and management. Field staff were trained in administering the questionnaire, interviewing techniques, and data entry on the Android app.

### 5.3. Pilot Testing

The questionnaires were pilot tested in 50 households to assess the feasibility of implementation and response times. The main objective of the pilot was to improve the language of the questionnaire, establish the order of questions, and check the accuracy and adequacy of the questionnaire instructions, such as ‘skip’ and ‘go to’. Clarity of instructions to the interviewers, respondents’ discomfort or embarrassment with certain questions, translation of technical terms, and the time needed to conduct an interview were also assessed. Further field challenges and the need for logistical arrangements were identified. The questionnaire and application were revised and finalized following the pilot test results and direct observations by survey supervisors.

### 5.4. Quality Assurance

Data were transferred from the handheld devices at the end of each day after synchronization and were transmitted directly to the AKU server. In remote locations with no internet access, the team leader manually exported a copy of the data to a Universal Serial Bus (USB) stick and saved it on a laptop to avoid data loss. The data collection application was password protected. Once the interview was saved, it could not be edited by the data collection staff. Data was encrypted to avoid breaches of confidentiality. The data were archived and stored in a data repository at Aga Khan University (AKU) in Karachi to which access was limited.

A Web-based RESTful secure Application Programming Interface (API) service was developed in Hypertext Preprocessor (PHP) to sync data from mobile devices to the server. A Microsoft Windows Server was used for hosting Apache Webserver and a MySQL database, which was securely installed on the AKU network. The database was backed up regularly to avoid accidental data loss. We also developed a web-based information portal using PHP and Google Charts library to visualize the collected data in real time. The portal had a comprehensive dashboard for real-time visualization, providing a snapshot of the activities of different teams and supporting survey data at clusters. Vaccination card pictures were taken by the data collectors and verified with the data entered.

Regular field monitoring and supervision were performed by the monitoring teams. Spot checks of data forms were conducted, and guidance was provided to the field teams. The community was not informed which day the data would be collected from their village, so it would not affect the spot checks for the sanitation index. The team also validated 10% of the data. The challenges faced by teams were discussed, solutions were developed, and feedback was provided to team leaders.

### 5.5. Statistical Analysis

We summarized categorical variables as frequencies and percentages. Normally distributed continuous variables were reported as means and standard deviations, while non-normally distributed continuous variables were reported as medians and interquartile ranges.

To understand the association of the intervention with the outcomes, the cross-sectional surveys were assembled into panel data sets and difference-in-differences (DID) analysis methods were used [36]. To estimate DID firstly, the differences between time points were estimated for the study groups, then the double difference was estimated across time points and arms. All the analysis was adjusted for the clustering effect.

A generalized linear model was used with standard robust variance estimation to consider the clustering effect. The binomial distribution was used with the Log link function to estimate risk ratios (RR) for binary outcomes while the Gaussian distribution was used for continuous outcomes. In addition, we used interaction estimators in unadjusted and covariate-adjusted regression methods to estimate the DID effect. The general model specification included an interaction term between the study period (baseline and endline) and intervention groups. Control variables were included as fixed or time-variant confounders measured at the individual or household level, including child age, gender, number of siblings, education and occupation of parents, household density and socio-economic status, and LHW coverage. A generalized linear model was fitted through ‘binreg’ and ‘glm’ routines in Stata software, version 17 [37]. Effect estimates were reported with 95% confidence intervals (CIs).

## 6. Discussion

The CoMIC trial was an evaluation of the effectiveness of a unique approach of collective community engagement and demand creation through community mobilization and incentivization for improving the uptake of essential interventions for the prevention and management of the two leading causes of under-five morbidity and mortality. To the best of our knowledge, this trial was the first of its kind to assess the effectiveness of unique conditional community-based incentives as an innovative strategy involving serial incremental targets of collective improvement in community behavior related to improvement in the coverage of a composite indicator of FIC, ORS, and SI.

The findings of this trial could suggest possible ways to influence positive behaviors that are difficult to change, but it is assumed that if a community works collectively, has autonomy over tasks, and is provided with tangible short-term incentives, there is a higher chance of bringing change in health behaviors even at the wider community level. The framework also assisted in identifying and targeting community perceptions regarding the significance of immunization, breastfeeding, ORS use, and other essential child health interventions.

Various forms of incentives, user-directed as well as provider-directed, have been previously assessed for their effectiveness in improving child health indicators in the literature and have shown some potential to improve coverage of child health interventions [15,16]. Cash transfers, microcredit, user fee removal, voucher schemes, and pay for performance are a few types of incentives utilized in health that provide direct or indirect monetary incentives and have shown the potential for improving participation in health education and attendance to healthcare visits coverage [15,17,18,19,20,21]. But this trial assessed a community incentive conditioned on a collective change in behaviors. The incentives were also decided by the community so that they could be more appealing and a cost-sharing model would ensure the sustainability of the incentives.

The CoMIC trial also utilized a composite index to measure household sanitation status through spot checks as composite indices are more stable over time compared with the individual indicators and are predictive of diarrhea-related events in young children [32]. Hence the CoMIC findings could also advocate the use of a composite index as a more stable and comprehensive assessment method for sanitation status for household surveys with spot checks being potentially a fast and efficient way of assessing hygiene at household levels. Ruel and Arimond suggested that spot checks may be less susceptible to variability because spot checks are designed to measure ‘proxies’ to behaviors rather than the actual behaviors themselves [38].

The collective nature of the intervention design in a closely interlinked society assumes that convincing and behavior change can be facilitated, and consequently, if the community at large is convinced, it becomes easier to enforce households within the community that are hesitant or reluctant for various reasons. This strategy, if proven effective, can be implemented on a larger scale. In the context of Pakistan, it can utilize the lowest administrative unit, which is the Union Council, which has a committee with an annual budget of its own and most of these funds are usually spent on providing WASH facilities. Hence, this strategy at a larger scale can not only ensure behavior change but also the proper utilization of funds at the local level, since it can involve participation from the local community.

## Figures and Tables

**Table 1:** Indices and items in the sanitation index.

Indices	Items
Drinking water index (DWI)	Interior water container water is covered
	Exterior water container is clean
	Container contains water
Food index (FI)	Clean dishes are covered
	Clean dishes are stored high
	All food is covered
Personal hygiene index (PHI)	Mother/caregiver is wearing shoes
	Mother’s/caregiver’s hands are clean
	Index child’s hands are clean
Domestic household hygiene index (DHI)	No trash in yard
	No trash inside home
	No unrestrained animals
	No dirty clothes accumulated in the home
	Insignificant quantity of flies inside the home
	No standing water on the home patio

**Table 2:** Local challenges and contextualized solutions.

Challenges	Solutions
Vaccination	
▪ Vaccinators do not visit villages on time to vaccinate the children. ▪ EPI center is not near the village ▪ Lack of transportation facilities to reach vaccination centers. ▪ Shortage of vaccine and non-availability of vaccinator at EPI facility	▪ VC members liaise with vaccinators to increase vaccinator visit. ▪ Schedule of the vaccinator visit date was shared with the community by VC members. ▪ VC members regularly checked vaccination cards for any leftover vaccine or child to ensure that vaccination is completed for each child. ▪ Group of people (5–6) rented a vehicle to reach the healthcare facility for vaccination as this was affordable and convenient for the villagers.
ORS	
▪ Unavailability of ORS at local shops in the villages.	▪ Ensured availability of ORS in local shop
Hygiene	
▪ Animals were kept in residential area. ▪ Lack of waste management and disposal. ▪ Cooked food was not kept in a safe and hygienic place and not covered properly. ▪ Clean cooking and serving utensils were not kept in a higher place and were not covered.	▪ Villagers constructed sheds (away from living space) for animals. ▪ Waste collection points were made in villages to dispose of waste. ▪ Villagers kept clean utensils covered and at height.
Breastfeeding	
▪ Mothers did not exclusively breastfeed their child for 6 months due to works in the field.	▪ Mother frequently visited home to breastfeed her child and if possible, avoided going to the field for work.

*Acronyms:* Expanded Program on Immunization (EPI); village committee (VC); oral rehydrating solution (ORS).

**Table 3:** Modules of the data collection tool.

Sections Included in the Data Collection Tool
Section A: Geo-Spatial location coordinates
section B: Household identification and demographic information
Section C: Introduction and consent
Section D: Household members’ information
Section E: Socio-economic status of household
Section F: Reproductive health, maternal health
Section G: Child health (diarrhea)
Section H: Child health (acute respiratory infection (ARI))
Section I: Immunization
Section J: Breastfeeding and nutrition
Section K: Water and sanitation
Section L: Handwashing
Section M: Sanitation index (spot check)

*Acronyms:* acute respiratory infection (ARI).

## Data Availability

This is just a methods paper and there is no data.
